# Editor's view: Value of information in the 21st century – examples from science, medicine, policy, media, and markets

**DOI:** 10.7189/jogh.15.01003

**Published:** 2025-06-20

**Authors:** Igor Rudan

**Affiliations:** 1 Centre for Global Health, Usher Institute, The University of Edinburgh, Edinburgh, UK; 2 Nuffield Department of Primary Care Health Sciences and Green Templeton College, Oxford University, Oxford, UK

## Abstract

This editorial explores the concept of the ‘value of information’ in the 21st century through five distinct domains: science, medicine, policy, media, and markets. It uses examples to show that not all information is of equal value. Valuable information shifts probabilities assigned to our hypotheses in the most meaningful ways. It significantly alters our knowledge and understanding of our context, helps us prioritise more rational ideas, and facilitates better decision-making. This paper also addresses the necessity of a conscious observer for the value of information to exist. It develops a framework for how the brain perceives, processes, and assigns value to new information. An example with childhood memories is used to explain the incremental and disruptive shifts in understanding that information of different value can cause. Further examples illustrate how well-designed research can amplify the value of information, how long-neglected information can be rediscovered and used to radically reshape policy, and how expert crowdsourcing can prioritise ideas and democratise decision-making. They also show how key pieces of the most valuable information can redirect policies, have large real-world impact, and save lives. The role of disinformation is also addressed, particularly its power to distort our shared understanding of the related context, mislead rational activities and prompt susceptible people to prioritise irrational ideas. Mainstream media and social media can enable rapid spread of disinformation, exploiting the greater intensity of the brain’s response to an unexpected surprise over the expected truth. Stock market prices, meanwhile, reflect in real time how each new information changes the perceived value of a company. Valuable information seems to have some inherent traits: relevance, *i.e.* it influences an observer's perception of the related beliefs or hypotheses; credibility, *i.e.* it needs to be trusted by an observer; and leverage, *i.e.* it influences an observer's ideas and decisions decisively. Where all three criteria align, information assumes a ‘high value coefficient’ and serves in revising prioritised ideas. Designing activities to increase this coefficient is therefore of common interest to scientists, health professionals, policymakers, journalists, and investors. As artificial intelligence accelerates the volume and speed of data production, human and machine systems alike will focus not merely on data collection, but on discerning which missing information, if generated, could enable the greatest positive impact on the real world. Ultimately, future success in science, medicine, policy, media, and markets may not necessarily be linked to gathering the largest amount information, but rather being good at identifying, generating, and acting upon the information that is most valuable.

Let us imagine the entire universe – the vast, chilling emptiness of space cloaked in darkness, where galaxies are scattered like islands adrift in a cosmic ocean, where stars glow as rare beacons of light and warmth, and where massive objects orbit alongside colossal black holes, surrounded by gravitational forces and swirling gaseous nebulae. In short, the universe in all its immensity.

Now, let us go one step further and imagine that this is not our universe, but some entirely different one. A universe wholly inaccessible not only to us, but to any other universe. A universe that, in principle, can never be reached, observed, or known by anything outside of itself. Finally, let us imagine one more detail: that in this unreachable universe, not a single conscious being exists. Only atoms, molecules, gas clouds, rocks… but nothing with even the faintest awareness of its own existence.

If we have managed to truly picture such a universe, a provocative question arises: does that universe even exist? If absolutely no one – and nothing, anywhere – cannot possibly know that this solitary universe exists, can we say it exists at all? And if someone argues that it does not, or that it makes no difference whether it does, then we can pose another question. Suppose that, by some means that we cannot yet understand, a single white mouse were dropped into this hidden universe, onto the surface of a planet where it could survive for a while. Is it possible that, with the appearance of this one tiny creature, this entire universe suddenly comes into being – simply because a mouse, with its five imperfect senses, became aware of it?

The moment it welcomes this unexpected guest, this universe would begin to offer an unfathomable wealth of information about itself to the mouse. But the mouse would be able to perceive only a minuscule fraction. Its senses – sight, hearing, smell, touch, and taste – would feed information to its brain, yet they have not evolved to understand the universe, but to help the mouse survive in its immediate environment. In the vacuum of space, hearing is useless, since there is no air to carry sound. Smell fares no better. Sight might still help, but the mouse – like humans – can perceive only a narrow slice of the electromagnetic spectrum. Taste is irrelevant in an interstellar space where there is no food, and touch is also meaningless when there is nothing to grasp or feel in the void.

Yet unlike the mouse or any other species that we know of, humans have succeeded in building instruments that vastly extend their perceptual reach. Through telescopes and microscopes, satellites and the Large Hadron Collider (LHC), humans can now glimpse distant galaxies and peer into the nuclei of atoms. Once humans built such tools that greatly extended their modest senses, the universe seemed quite happy to reveal a variety of information about itself to scientists. Those scientific tools allowed humans to explore the universe and not merely exist or survive within it. This is a staggering achievement for tiny creatures that humans are. Among the billions of species that have evolved on Earth over billions of years and the roughly ten million that still remain, only humans seem to have developed such tools [1]. All other species were living and dying throughout the history of the planet Earth, without any hope of understanding of the world they found themselves in. That mere thought is slightly scary – can humans living in the 21st century really be that lucky?

It is also fascinating that not all the information we gather holds equal value in helping us understand the world around us. Some pieces are like keys that unlock entirely new insights, fundamentally altering our previous understanding; others merely confirm what we already know or what we suspected. Here we encounter the curious relationship between our brain, the central processor of all incoming sensory information, and the information itself. Faced with an overwhelming flood of information and data, the brain gradually learns to ignore what is familiar, expected, or redundant. It filters out the known to conserve energy and focus.

In this flood of information, the brain remains ever-vigilant, restlessly scanning for anomalies, for the rare signals that contradict its expectations. These are the moments that matter most. They force the brain to revise its model of reality, to reshape its understanding of the environment, and in doing so, they push us forward, one small insight at a time, toward a deeper understanding of the universe we inhabit.

By this point, we should try to answer the seemingly simple question – what is ‘information’? The anthropologist Gregory Bateson notably defined it as ‘a difference which makes a difference’ [2]. This is a refreshingly clever definition of something that is quite difficult to define, but it also implies something important: the presence of a conscious observer. From this definition, in order to exist, any ‘information’ would need at least one observer – a conscious being for whom the observed difference would ‘make a difference’. That difference would arise from observers' perceptive abilities, memory, and expectations based on their internal models, thus necessitating some degree of consciousness.

## THE BRAIN’S PERCEPTION, PROCESSING, AND VALUATION OF INFORMATION

Now that we have a working definition of the term ‘information’, we should ask another question: what makes information ‘valuable’? To illustrate how the brain perceives and processes information and assigns value to it, I will offer a personal example. Imagine my sister and I are having lunch. She suddenly asks: ‘Do you remember if you liked yellow rubber ducklings when you were a baby, lying in your cradle?’ Clearly, I do not recall – maybe I did, and maybe I did not. With no related memory, no information to use to guide my answer, I can only assign equal weight to both possibilities – a 50% chance either way – and keep that neutral assumption until any new information comes along to affect my belief.

Yet, we all carry something else – our intuition. That subtle, persistent expectation based on the vast amount of information stored away in our subconscious. These intuitive prompts might arise from patterns our brains have processed continuously over many years, even if we do not consciously recall them. So, if I had to rely on nothing but intuition to guess whether I liked yellow ducks as a baby, I would lean toward ‘probably not’. I had no particular fondness for ducks in my adulthood. Based on that vague inner feeling, I would get a tendency to revise my estimate: perhaps only a 20% chance that I liked yellow ducks as a baby and 80% that I did not. This deviation from 50:50 is my personal, inherent bias, and there is nothing rational about it.

But then, let us say I visit my elderly grandmother at her nursing home. After chatting about her health, I bring up my sister’s question. My grandmother was around when I was a baby – maybe she still remembers. When I ask, she smiles and says, ‘Oh yes, I think you really liked yellow ducklings when you were a baby!’ Now, that is new information that she just created for me. It is relevant, my brain now has to process it, evaluate its credibility, and update my belief accordingly. My grandmother is well over ninety, so her memory might not be reliable. Perhaps she did not hear me well, as her hearing is poor, too. Still, she did create new information and, as much as it goes against my intuition and personal bias, I cannot disregard it anymore. So, I might need to increase the probability that I liked yellow ducklings as a baby from my preferred guess of 20% to around 40%. This is, again, not very rational, because it shows that my trust in my own intuition remains greater than my confidence in my grandmother's fading memory and hearing.

Then, let us suppose that I go to lunch with my mother and ask her the same question. Her memory is sharper than my grandmother’s, even though it has been more than five decades since those times. She replies without hesitation: ‘Yes, you definitely loved those yellow ducklings.’ Now, my mother created another piece of information. I value it more than the first one. Taking into account her statement, my grandmother’s recollection, and my intuition, I now feel compelled to revise the probability that I liked yellow ducks once again. This time, I will need to increase it quite significantly: from 40% to, say, 90%.

Then, my sister joins us. She has been digging through old family albums, she says, and found a photo of me as a baby in the cradle surrounded by as many as three different yellow ducklings. She has taken a snapshot of the photo on her phone and shows it to me. Sure enough, there I am as a baby, in our old flat, with three unmistakable yellow ducks nearby. This feels like definitive proof. My parents were always modest; they would have never bought so many duck toys for me unless I really adored them. Convinced, I adjust my probability once more – this time from 90% to 99%. One should never go all the way to 100%, I think to myself. We just never know how much we still do not know. There is always the slimmest possibility, signified in that 1%, that I reserve for some bizarre coincidence or an entirely unforeseen explanation.

Finally, my father arrives for lunch. I decide to ask him too, just in case, if he also remembers whether I liked yellow ducks as a baby? Suddenly, he bursts out laughter: ‘Your sister just learned how to use Photoshop,’ he tells me. ‘She has been editing old family photos and adding things into them for fun. She inserted those three ducks into a photo of you in the crib and then got your grandmother, mother, and me all engaged in this practical joke to convince you that you had been a yellow-duck-loving baby.’

This new piece of information from the father instantly unravels the most likely truth. My 99% certainty plummets, now almost all the way down to zero. But never to zero, as I now hardly believe anyone anything. Still, I know that my father does not have it in him to try to confuse me or to trick me like this. Yet despite trusting him fully, some doubt does creep in. After all that back-and-forth, I no longer feel completely sure of anything. I revise my ‘knowledge’ on this question yet again: I will consider the probability that I liked ducks as a baby to be only about 10%.

Now, let us step back and examine this little journey through the lens of information perception, processing, and the value assigned to each new information when testing a new hypothesis, in this case: ‘Did I like yellow ducklings as a baby?’

I started with no relevant information, so I needed to give equal probability – 50% – to both outcomes. Then, intuition and personal biases led me to lower that to 20%. So, a 30% downward adjustment was based purely on a vague inner feeling. My grandmother’s recollection nudged it upward by 20%, but the information based on my mother’s memory was more ‘valuable’, causing an upward shift by 50%. My sister’s apparent photographic evidence seemed definitive, raising it by another 9%. However, that final stretch, from 90% to 99%, required an exceptionally high degree of confidence. Typically, it is very hard to close that gap toward almost-certainty, unless we can generate truly strong evidence.

Then came my father’s revelation. His information was not only the most ‘valuable’, causing a downward shift of as much as 89%, but it was also disruptive and transformative. It did not simply reduce the probability of the hypothesis that I accepted from 99% down to 10% – it also explained why all the prior information had misled me. That is a crucial function of the information of the highest value: it does not just shift prior beliefs radically – it can also entirely rewrite the context. It enables us not only to change our minds, but to understand why we were mistaken in the first place.

## SCIENCE'S CREATION OF NEW KNOWLEDGE THROUGH GENERATION OF VALUABLE INFORMATION

The way our brain perceives and processes information is not just a curiosity of individual psychology. It mirrors the very mechanism through which all human knowledge evolves through scientific research. Contrary to what many in the population may believe, scientific knowledge is not a rigid collection of eternal truths. Instead, it can be best understood as a vast and dynamic landscape of probabilities assigned to different hypotheses, as it was demonstrated in the above example. It is continuously updated ‘map’, reflecting how certain we are about any given assumption about our world, based on all the research and experimentation conducted and all information generated to date.

There are things we know with near-absolute confidence. The certainty in the association between some human genetic variants and specific diseases, for instance, can now be expressed with a probability of 99.999999999…%, *i.e.* a figure with more than two hundred ‘nines’ after the decimal. Our confidence in the existence of the Higgs boson particle stands at above 99.999999% [3]. Yet in other areas, our certainty is comparatively much lower. Take, for example, climate change. During the first decade of the 21st century, based on all the useful information generated at the time, climate scientists were becoming about 90% sure that serious changes were underway and that they were largely caused by human activity [4]. That figure varied slightly, depending on which data were prioritised. Although quite high, such a probability made it difficult for scientists to persuade policy makers and the public. Sceptics could still cling to a sufficient level of uncertainty. But as new information was steadily added over the next several years, the probability and the consensus grew increasingly stronger, rising to 95% by the year 2013 [5].

To make meaningful contributions to science, it is advisable for future researchers to first develop a ‘feel’ for the value of the information that will be produced by their scientific work. This starts with understanding the current landscape of probabilities assigned to different hypotheses within the field of interest, *i.e.* the extent to which each hypothesis is either supported or questioned by existing information. In areas where we know nothing, where no data exists, every hypothesis starts at 50% probability of being true, because it could be either true or false. In more developed areas, probabilities may span the full spectrum from near-zero to near-certain, depending on the depth and quality of information available to test them experimentally.

This is the key building block of a successful scientific strategy. Once we understand where the probabilities currently lie, we can better judge to where new information that we could generate might shift them in meaningful ways. In areas already saturated with knowledge, further information will often yield diminishing returns. However, in areas of uncertainty or disagreement, a well-designed study can produce information that would move the probability to a very high threshold, and with it, the frontier of science.

That last sentence may need some reflection. It explains that, in scientific research, it is not only important to create relevant new information, but to design the study in a way that makes the resulting information credible and firm. This is especially important when attempting to close a major gap in understanding or to challenge a widely held belief. The value of information will surely depend on its source and the way in which it was generated. But if the rules of scientific research were closely followed, then all information generated by research should be credible, and its main value will depend on its ‘leverage’ – *i.e.* how will it impact the present spectrum of probabilities assigned to different hypotheses.

Let us consider what kind of information will truly drive science forward. For example, when we know absolutely nothing about a subject, all competing hypotheses are equally probable. If a new study generates credible information that increases the probability of one hypothesis to close to 80% and reduces the probabilities for all the others to single digits, that is a major leap. It represents a significant scientific discovery. If a subsequent, more rigorous and better-designed study confirms those findings and increases the probability to 99%, that is an even more impressive contribution, one that may earn high recognition or an award.

Yet science also prizes, perhaps even more so, a different kind of breakthrough: the disruptive discovery. That is a finding that often arises unexpectedly and then, once verified, causes a dramatic shift in understanding, reducing the probability of a previously dominant hypothesis from 80–90% to near zero. Such disruptions open new avenues for exploration and reset the trajectory of entire research fields. Their value for science is immense, because they do not just add to knowledge, but also correct long-standing mistaken paths of progress. They reveal that science, for all its strength, had been betting on the wrong hypothesis, and now it finally knows better.

The implication is clear: high-impact science often happens in three spaces on this spectrum of probabilities. First, where no information exists yet and the first information is generated through pioneering research, thus moving the probability for one hypothesis upwards, and all other downwards – that is ‘the pioneering progress’. Second, where existing certainty is already quite high, say 80–90%, and new information produced by well-designed research pushes it to near-absolute levels, like 99.999%. Third, and perhaps the most valuable to science – because it is not possible to achieve this result through a well-designed science alone, like the previous two cases – in rare occasions where a hypothesis widely believed to be true is brought back down to near zero probability, opening unexplored new paths to scientific truth. These are the zones on the probability spectrum of real transformation for scientific fields.

By contrast, research that nudges probabilities only slightly – say, from 65% to 70% – is unlikely to have similar impact, even if it is methodologically perfectly sound and intellectually honest. Such studies play an important role in building the overall body of knowledge, laying down incremental bricks towards a larger accomplishment. But until those bricks accumulate into something striking, their individual value will rarely be seen as revolutionary. Similarly, working in domains where confidence is already near total, say, 95% or more, is not a fruitful path for a scientific career. There is little room left to alter the probability in any meaningful way. No matter how meticulous the effort, it cannot create the kind of valuable information that reshapes the existing human knowledge. Understanding where to invest our attention and how to construct research that would derive its value from having a chance to meaningfully alter the existing probabilities for the tested hypotheses is what distinguishes routine work from truly impactful science.

In my previous paper, I explained the brain's underappreciated sense – its ‘perception of ideas’ [6]. Here I propose that, in addition, the brain can also generate a sense for the ‘value of information’. In the next section, I will draw from my own experiences – in genetic research, global health, influencing policies, working with media, and observing financial markets – to illustrate how this ‘sense’ of ideas, combined with a ‘sense’ for the value of information, has guided my own ideas throughout my career as a scientist.

## VALUE OF INFORMATION: AN EXAMPLE FROM SCIENCE (GENOME-WIDE ASSOCIATION STUDIES OF COMPLEX TRAITS AND DISEASES)

When the Human Genome Project reached completion, global scientific attention turned immediately to a key question: what is the genetic basis of human diseases? The next step seemed obvious. To find the genes associated with human diseases, it was necessary to assemble a few thousand people diagnosed with a particular disease, along with a few thousand healthy controls, and determine genetic variants in their individual genomes. Then, by comparing these two groups of genomes, researchers should be able to identify genetic variants that are more commonly found in the group of people who have the disease [7].

This study design made sense. But let us reconsider it from the perspective of the ‘value of information’. At the dawn of the 21st century, performing genome-wide scans in thousands of genomes came with a large price tag – millions of dollars were needed. Clearly, in this case, the information stored in genomes of particular humans had a value that could even be expressed in financial terms. What would this investment yield? At best, it would produce a list of genetic variants associated with a single disease, shared among the first group and relatively absent in the second group. However, we also knew that diseases are almost never caused by genetics alone. They are shaped by a complex interplay of environmental, cultural, behavioural, psychological, and other factors. Moreover, we could not have known how many genes might be underlying a complex human disease: were we looking for only one or two, or perhaps dozens, hundreds, or even thousands?

Working on this problem with two colleagues in Edinburgh, Professors Harry Campbell and Alan F. Wright, it became apparent to us that this financial investment in genomic information could be approached differently. Rather than focussing on a single disease, why not create a resource of thousands of individuals, regardless of their disease status, in whom we could study hundreds of quantitatively measurable traits in the human body? Most of those traits have already been associated with some diseases once their values depart from normal ranges, thus indicating decompensation in underlying physiological mechanisms. We could study truly diverse traits, from weight, blood pressure, and pulse, to visual acuity, skinfold thickness, sleep patterns, and cognitive ability. From kidney, liver, and lung function to a rich spectrum of metabolites, enzymes, and proteins circulating in the blood. If we found how those quantitative traits are controlled by the genetic variants, then we could conduct additional, much smaller and simpler studies. In them, we could test if the identified genes can also be associated with many diseases already known to be related to those quantitative traits. But, by focussing on hundreds of quantitative traits instead on only one disease, and for the same amount of financial investment (because several thousand genomes needed to be scanned in both approaches), we could use that genomic information to address hundreds of interesting questions instead of just one.

We developed the plan further, based on another critical insight: to detect the effects of individual genes with clarity, we had to reduce the ‘background noise’ of both genetic and non-genetic effects. That could be done by choosing a very specific population that would be homogenous both genetically, culturally, and environmentally, so that ancestry, lifestyle, occupation, nutrition, and environmental exposures did not vary much from person to person. In such a dataset, it would be much easier to isolate the signal from the noise.

That is when the Croatian islands in the coastal region of Dalmatia emerged as an advantageous research setting. Their isolated highland populations, with low genetic diversity, genealogies available for up to seven ancestral generations, low immigration rates, high levels of consanguinity, typical occupation in agriculture, and shared environmental conditions over the entire lifetime offered an unparalleled opportunity to explore the genetic basis of human biology and disease. That is how the large research programme ‘10 001 Dalmatians’ was born [8].

Why was the value of information on genetic variants in several thousands of Croatian islanders so much greater than that from the hospitals in much wealthier countries? Because the same investment spent on scanning genetic variants among thousands of people in genetically, culturally, environmentally, and socially diverse Western urban populations could, at best, yield a few possible associations between genetic variants and a single selected disease. Even that result, if achieved, would be attenuated by an overwhelming amount of ‘noise’ arising from individual variability. In contrast, Croatian island studies could identify genetic variants involved not in just one, but in hundreds of human quantitative traits. Their identification would allow us to start building our understanding of how groups of genes work together in normal physiology and how disruptions in those networks lead to human diseases.

These changes in study design multiplied the potential value of genomic information hundreds of times in comparison to case-control studies of single diseases, at least in principle. Clearly, it is not possible to know in advance if a single discovery underlying a single disease would eventually become more important and commercially exploitable than many findings relevant to quantitative biological traits. However, in the absence of any information, the approach used in the ‘10 001 Dalmatians’ study has maximised the chances to obtain many interesting findings. By increasing the signal-to-noise ratio and focussing on many traits at once, we eventually identified more than 2000 genetic variants involved in a wide array of biological functions and disease processes. That number is still growing almost 20 years since our first results, while most of single disease studies were exhausted after the first analyses were completed. The research model created in the ‘10 001 Dalmatians’ project transformed a standard genetic epidemiological study into a goldmine of valuable information, attracting significant international research investment.

Ultimately, what made this possible was following a simple, yet well-informed idea: to carefully design the study and expand the scope of research to massively amplify the value of each generated piece of genomic information. It is an example of how a human ‘sense of ideas’, described in my previous paper [6], responded positively to a possibility of increasing the value of generated information by changing the design of the study.

## VALUE OF INFORMATION: AN EXAMPLE FROM MEDICINE (EPIDEMIOLOGICAL ANALYSIS OF THE CAUSES OF GLOBAL CHILD MORTALITY)

At a certain point of the ‘10 001 Dalmatians’ project, I came to a sobering realisation. The genetic architecture of human quantitative traits and common diseases was so intricate and overwhelmingly complex that translating the many fascinating discoveries we were making into rapid, practical medical applications would be far more difficult than I had hoped. I felt like we only managed to expose a new layer of remarkable complexity, but without much hope of translating those insights into better health of the population. While my fellow geneticists and bioinformaticians were still – and rightly so – captivated by the intellectual thrill of scanning the human genome and understanding what it does by associating variants to various human traits, I began to feel restless. As a medical doctor, I did not just want to understand human life better – I wanted to improve it, too. It became apparent to me that all the individual effects from specific genetic variants were miniscule, even in such a well-chosen population as the one we had. I could not easily see how would the remarkable genetic diversity and complexity that we were exposing be used to practically cure patients that are ill, or to prevent the diseases from developing. I wanted to take part in scientific efforts that could be translated into tangible gains for human health, particularly at the population level.

That is when I began to gradually step back from the intensity of genomic research and started to reflect more broadly: where, exactly, were the largest gaps in our knowledge that could make a difference to the population that presently lives on Earth? What was the key missing information, which would be most valuable to have? Where were the most obvious blind spots that stood in the way of meaningful progress for the 21st-century medicine?

While considering what the best answers to this question might be, I thought that understanding the United Nations’ Millennium Development Goals (MDGs) could be useful [9]. One fact leapt out from studying the MDGs: at the dawn of the 21st century, an astonishing eleven million children were still dying every year in low- and middle-income countries. That fact seemed incomprehensible to me. In high-income nations, child mortality had been reduced to a tiny fraction of that number. Why was such progress not mirrored elsewhere in the world? If we know how to prevent deaths in pre-school children in Finland, Canada, or Japan, why are we not doing the same things everywhere else?

I soon found the answer to that question: in the year 2000, hardly anyone dared to guess what the causes of child mortality in low- and middle-income countries of the world may be. Those countries were simply too poor to carry out even a census of their population regularly, let alone to establish causes of death for every person who dies. This is especially true when deaths occur outside of the reach of those countries’ underdeveloped health systems. Most child deaths were occurring exactly in those places, where no health care was available.

This was startling. The main reason why child mortality remained so high was surprisingly basic: no one really knew, with any precision, what children were dying from. Without reliable data, the world was essentially fighting a war against child mortality blindfolded. Resources were being poured into diseases that were possibly not even among the leading causes of death. This situation resembled one of the previous examples on the ‘value of information’: the case where no reliable information exists and the first clues still need to be generated, making such information truly valuable – particularly in the case of saving the small children globally. So, to find answers, I joined the Child Health Epidemiology Reference Group (CHERG) with my colleague from Edinburgh, Professor Harry Campbell. This was an expert advisory body to the World Health Organization and UNICEF. Our mission was simple in principle, yet daunting in practice: to identify and rank, by their relative importance, the causes of child mortality around the world.

We turned to research again, but not some modern, high-tech science. We immersed ourselves into something closer to ‘archaeology of valuable information’. We began to sift through the medical literature, scouring long-forgotten papers, hidden in dusty archives or obscure journals. They had been written by exceptionally rare doctors who had spent years, even decades, working in remote villages across Africa, Asia, and Latin America. These were physicians who had observed and recorded, often with great care and diligence, the illnesses that claimed children’s lives in these underprivileged settings. We screened tens of thousands of papers that were published over the 40-year period between 1961 and 2000.

Bit by bit, we reconstructed a mosaic of information on the causes of child deaths globally. When the fragments came together, an astonishing picture emerged: nearly half of all child deaths were due to just two causes that were not suspected as being nearly that important: childhood pneumonia and diarrhoea. This information was particularly difficult to take in, because both diseases were highly preventable and treatable: effective vaccines, inexpensive antibiotics, and oral rehydration therapies already existed. Millions of lives could have been saved had we only known where to direct our efforts.

This was the concept of ‘value of information’ at its most powerful. By gathering and synthesising forgotten fragments of information that were sitting silently in many different journals and were long forgotten by the research community, we had created new, highly valuable and actionable mosaic of information. In doing so, we succeeded in making the invisible visible. Before the CHERG's work, pneumonia and diarrhoea were not near the top of policy agendas. They were not prioritised in national health plans, few people studied them, and they were barely mentioned in funding proposals. Because of this, they were often overlooked by international donors [10]. Yet once it became clear that these two conditions alone were responsible for such a vast number of child deaths, everything changed. New, powerful international coalitions were formed between the donors, the international organisations, the governments, and the industry. Large innovative programmes were launched, such as the ‘Advance Market Commitment’ [11]. Prices of vaccines and antibiotics were successfully reduced for their implementation in poor countries and they were widely distributed. Awareness campaigns were funded. Simple treatments were scaled up. The number of child deaths was reduced by about a half within the next decade, saving millions of lives in the poorest countries each year. In this context, the value of the information that CHERG produced did not just fill a gap in academic knowledge – it shifted global priorities, saving millions of children’s lives as a result.

## VALUE OF INFORMATION: AN EXAMPLE FROM POLICY (SETTING INVESTMENT PRIORITIES IN GLOBAL HEALTH AND DEVELOPMENT)

In the world of international health and development policy, one fundamental challenge stands above all others: given the abundance of investment opportunities on one hand and the overwhelming number of global health and development problems on the other, how do we decide where to invest? This question becomes even more complex when we realise that priorities must constantly be recalibrated at three different levels: investment in research (to generate new knowledge and innovation), investment in interventions (to assist those already suffering), and investment in development (to prevent or reduce exposure to risks). Yet despite its urgency, no simple, democratic, transparent, or universally acceptable system existed to guide such prioritisation.

I began to reflect on a deeper question: what kind of information would actually be ‘valuable’ for setting such investment priorities? If such information did not exist, as in the previous example, how could we generate it ‘from nothing’ yet again?

I found myself in many conversations with academics, policymakers, and even investors in health and development, all of whom were eager to set priorities, but uncertain how to do it. It is one thing to declare that a particular investment area is important; it is another to back it up with credible, evidence-based reasoning. After all, outcomes in global health and development are notoriously difficult to predict. How can one assess an endless stream of ideas that all seem important and require funding systematically and fairly?

I was thinking about this problem during the first decade of the 21st century, a period defined by rapid technological shifts: the rise of digital networks, online collaboration, and a growing movement that was later named ‘crowdsourcing’. Large quantities of useful information, knowledge, or even mere opinions could be collected from a wide pool of people thanks to the internet. This could suddenly be done online, quickly and cost-effectively. I realised that this presented a unique opportunity. What if we could gather insights on all those many ideas from hundreds of the most knowledgeable people in the world at the same time?

Importantly, each of these chosen experts carried within them vast stores of knowledge and experience that could be refined into expert intuition about what is attractive, feasible, and impactful – therefore, meaningful and worthwhile – using the basic criteria for assessing ideas that I described previously [6]. If we could tap into their collective brainpower, ‘connect’ their brains through a crowdsourcing process, and ask them to contribute their best ideas on how to solve a major global health or development problem, we could then organise those ideas into a structured framework. For each idea, we could define its core components: what would be needed to take it from conception to implementation?

If we then asked those same experts to score each idea across those components – evaluating, for instance, its likelihood of answerability, feasibility, scalability, potential impact, or likely effects on equity – we would suddenly have a way to visualise expert consensus. Crowdsourcing would turn hundreds of scattered judgments into one coherent, evidence-informed picture.

This is how the Child Health and Nutrition Research Initiative (CHNRI) methodology, named after the group that hosted its development, was born and presented in 2007–08. I developed this framework with assistance from a larger transdisciplinary team to help set research priorities in global health and development, and in biomedicine quite generally. It already became the most widely used method of its kind over the following decade, gradually replacing previous approaches [12]. Its successful adoption and widespread use can be attributed to several of its features. The most important is that it produces large volumes of high-quality, actionable information, in a quick and inexpensive way, through broad expert participation. Therefore, it is seen as democratic, because no single individual’s input can have large influence on the final outcome. It is also transparent and replicable, and the output is simple and intuitive: a large table in which each proposed idea receives scores against each of a set of pre-defined criteria that can make it ‘better’ or ‘worse’.

In this way, the CHNRI method basically ‘measures’ the potential of many different ideas using collective optimism of a large group of experts in the subject [13]. Their scores are turned into values that range between 0% and 100%, so they are highly intuitive to the users of this generated information. This allows funders and policymakers to see far beyond the limits of any single individual’s expertise, as well as any single idea – they can see the full spectrum at once and compare the ideas against each other in their strengths and weaknesses, as judged by the most informed experts. So, the CHNRI method generates remarkably valuable information for funders and policymakers from, quite literally, ‘thin air’ – by simply asking a group of experts about their best ideas and then letting them all score those ideas against pre-defined criteria, which allows us to identify one idea as ‘better’ that another.

Building on this approach, I also helped develop the Equitable Impact Sensitive Tool (EQUIST) and the Pathways to Survival (PATHS) tool. They are used to guide investments in health interventions, particularly in low- and middle-income countries. Both of them, but particularly the PATHS tool, are based on the ‘value of information’. The PATHS tool, for example, visualises the paths that an individual can take, based on their context, towards a positive or a negative health outcome, and how additional information at each step could push them towards the path where survival is more likely. Today, these methods are routinely used by governments and global health agencies to inform investments into very large funding programmes that aim to improve health outcomes for the most underprivileged. All these tools were made possible by a deep understanding of what makes scientific information valuable.

## VALUE OF INFORMATION: AN EXAMPLE FROM THE MEDIA

Let us now step away from the world of science and enter the wider society – a space increasingly shaped not just by information, but by disinformation. In recent meetings, the world’s political and economic leaders have reached a sobering consensus: disinformation may well be the leading threat of the 21st century [14]. This presents us with a new challenge. We now have some understanding of what information is and some sense of what makes it valuable. But what exactly is disinformation and why does it matter so much?

To answer that, we must return to my previous paper and understand one of the brain's core functions [6]. It is, in essence, a device for perceiving, assessing, prioritising, and reacting to ideas. But it does not do so in a vacuum – it responds to ideas within a context. In an example given in the previous paper [6], when we are driving and another car is coming towards us, threatening a head-on collision, the idea of swerving off the road becomes not just reasonable, but life-saving. In that context, it is the best possible idea. But in the absence of oncoming traffic, the same idea is the worst possible – it is life-threatening for absolutely no reason. So, when prioritising ideas, understanding the context quite well is incredibly important.

Here is why disinformation is so dangerous: because it distorts the understanding of the context for everyone exposed to the falsehood that is spreading. It gets injected into our understanding of reality, making us more likely to adopt poor judgments, follow unreasonable ideas, or act against our own interests and the interests of the society as a whole. Whoever creates and spreads disinformation is degrading the very quality of public reasoning. From a scientist’s perspective, this is a uniquely frustrating challenge to address. Historically, in the mid-20th century, when scientists produced evidence relevant to public health or safety, they could communicate their findings to politicians, who would then work with the media to inform the public. That model functioned well through the 1950s and 1960s. People learned how to take medicine correctly, how to build safe infrastructure, how to follow science-based guidance. Many terrible diseases were eradicated or brought under control all over the world during those two decades. But then, things changed.

As multinational corporations rose in power, gradually eclipsing even some governments, they began to shape the public discourse. When scientists first reported that smoking was harmful, the tobacco industry responded not with reflection, but with resistance. With immense wealth and influence, they launched many activities to cast doubt on the research that produced the results that were threatening their businesses [15,16].

The same story repeated itself in the 1980s and 1990s, when climate scientists warned of global warming and climate change. This time, it was the oil and gas industry that felt threatened; science was again undermined in many ways, and the truth hidden from the public [17]. Through it all, the media – who do not exist in a vacuum, but rather depend on advertising revenue from those very industries – were rarely able to act as a neutral or independent mediator.

Then came the COVID-19 pandemic. Scientists offered evidence-based recommendations to save lives and reduce suffering, while being mindful of the economic costs. But their recommendations conflicted with the interests of many industries, ranging from fashion to retail, restaurants to sports, airlines to cruise ships, entertainment to hotels, weddings to gambling. Once again, the scientific voice was drowned in a sea of vested interests, manipulated information and disinformation, and manufactured doubt [18,19]. Media professionals are in an unfortunate position in such situations, because they are trained to always present different views on any story and let the audience make up their mind.

But, in some contexts, knowledge is the only currency. An example is a ship captain's knowledge guiding a cruise ship with thousands of passengers through an unexpected, major sea storm. In those cases, the captain was trained what to do, based on decades of experience and research, and the passengers on the ship have no useful knowledge to contribute. Only the captain's opinion on what needs to be done is relevant and credible, while an opposing view of a random passenger who feels sea-sick and uncomfortable and thinks that ship should be operated differently is entirely irrelevant. If promoted to all passengers equally as the captain’s opinion, this would make captain’s work much harder, and it could be dangerous for everyone on the ship.

Those who were engaged in COVID-19 response, especially if they worked with the media, are now the third generation of scientists facing this challenge. Scientists do not have the resources to match the communications machinery of multinational corporations. Their only position of power is speaking the scientific truth, which is rarely exciting or desired. Disinformation, on the other hand, surprises people. It excites and entertains them. It spreads much more quickly and widely than information. Learning from the earlier example of the yellow ducks, it will always be more captivating to hear that a scientist was mistaking, or deliberately lying, or that they may be corrupt, or secretly manipulating data. That information will spread very fast, because it defies people's expectations. The damage it does, however, is immense.

What can scientists do? In many cases, sadly, very little. It took decades for the public globally to finally accept the scientific truths on smoking. In the meantime, tens of millions have died due to smoking [20]. Today, we are preparing for climate change. The fires and floods we are witnessing in the past years are no surprise to science, they were predicted decades ago. But action was delayed, postponed, and even obstructed [17].

This is not just a struggle of scientists. It is a struggle for society as a whole. Disinformation threatens us all. Therefore, I believe that scientists should still do what they can to popularise science, write books on science for the public, produce documentary series, attend public festivals on science, and speak to the media wherever this is safe enough for them. In the face of disinformation, the quiet, steady voice of scientific truth should continue to speak and it needs to be heard. Because, once released, the truth tends to find its ways to survive.

A new scientific discipline is now emerging, one focussed on understanding and combating disinformation [21] and especially its spread through social media. Just as the first guns equalised the battlefield between the valiant and the vile, social media has equalised the promotion of useful information and complete nonsense, with profound consequences – primarily, for the brain's ‘sense of ideas’ [6]. These platforms, unlike traditional publishers, remain largely unregulated. Meanwhile, we have entered what could be called the ‘post-state’ era where social networks and some media outlets enable construction of false realities, with a common theme in undermining multiple social contracts that exist within the society and keep it more stable. As a result, the world is becoming an increasingly unpredictable place. Yet we have all built it together, so now we must find the wisdom to navigate it.

## VALUE OF INFORMATION: AN EXAMPLE FROM MARKETS (PREDICTING THE FUTURE WITH INFORMATION)

We might now ask a new question: can we use information to predict the future? Is it already written, pre-programmed, and we simply lack enough information to foresee it? In some domains, the answer is clearly – ‘yes’. For instance, we can predict with remarkable precision where the Moon will be relative to the Earth on a given date, say, 300 years from now. How is that possible? Because we already know the values for all the key variables and understand all the relevant relationships. The Earth-Moon system is simple enough, well understood, and all the key forces and the parameters are known. In such cases, the prediction of the future is not ‘magic’ – it is just the matter of reasonably simple computations.

This leads to a tantalising thought: could everything in the universe be just as predictable, if only we had access to all the necessary parameters? Our brains do not have that power, but that question may, perhaps, be partly answered by artificial intelligence (AI) at some point in the future. AI algorithms can already process volumes of data far beyond the ability of the human brain to analyse. If they begin predicting events with uncanny accuracy, we probably will not be able to understand how they arrive at their conclusions. Understandably, that is somewhat unsettling.

Meanwhile, the amount of data stored in computers continues to grow exponentially. This means that a point will be reached when we will be adding more digital data each year than had been generated in the entire previous human history. With such explosive expansion, we may eventually reach some truly important threshold, a critical mass of information sufficient to understand – and perhaps cure – almost any disease. At that point, two things will be important: whether we are still alive and whether we can afford the treatments that would suddenly become available to us. This is a good argument for trying to keep healthy and accumulate wealth, as the future is becoming truly unpredictable – incredibly positive things may happen, too, and not just the unwelcome ones.

The mention of accumulating wealth reminds me of an event where I gave a public lecture on the ‘value of information’. A member of the audience then stood up and suggested that I touched upon just about everything, except what really interested them: how could they become wealthy using the ‘value of information’ concept? I thought it was a fair question, and one that science could answer. If we consider the stock market and take a look at any chart of share prices, we will see the erratic dance of uncertainty. These movements in price reflect, in real time, how each new piece of information changes the perceived value of a company. A single positive announcement and the stock jumps. One bad headline and it plummets. Day in, day out, the price of the stocks never rests.

So, anyone who understands something about any product or a company that others do not should possess valuable information. If anyone has information that can help them foresee the future of some product before others do, that information is valuable and they could act on that insight. In that sense, valuable information can make people wealthy in a most direct way – through investing in stocks of the companies that will benefit from successful new products or services.

## CONCLUSION: THE ARCHITECTURE OF VALUABLE INFORMATION

Lastly, let us address the larger question: how does information relate to the totality of human knowledge? Essentially, human knowledge is the set of all probabilities that we collectively attribute to all possible hypotheses, in light of all the information available to us. Scientists, but also everyday observers, create this knowledge through constant interaction with reality and perception of the information that it provides.

Across science, medicine, policy, media, and markets, one pattern emerges: information is the substrate of our individual realities, the unseen architecture that frames our beliefs, behaviours, choices, and our likely futures. A single principle seems to unite these diverse examples: the most valuable information is that which shifts probabilities the most in a meaningful way. It clarifies a blurry view, challenges a dominant narrative, or opens a path that was previously unseen. The resulting shift in probabilities, whether upward or downward, transforms not just our knowledge, but also sharpens our perception of ideas, thus inspiring and informing our future actions. In each example, we could note the brain’s capacity to assign value to information within any given context, and then to reassess and recalibrate this value. We now live in age when we are overwhelmed by abundance of information – something to which we have not had an opportunity to adapt during our evolution. Our new challenge is how to discern fragments of the informational noise that are valuable.

All five examples in this paper demonstrate that valuable information almost always shares a few defining traits. It emerges from contexts of uncertainty, where competing explanations or decisions exist. Then, it reduces uncertainty in a meaningful way by recalibrating probabilities in the mind of the observer. It must be trustworthy, and it must illuminate, making apparent what was hidden, and knowable what was previously unknown. These examples also show that information, when truly valuable, is rarely incremental. Instead, it is catalytic, moving us to a different ‘state’ of knowing, explaining why we were wrong before and what should we do now. It updates our prior beliefs, reshapes our narratives, and reorders our prioritised ideas. This is why disinformation is so dangerous: by inserting falsehoods, it degrades our ability to judge, to decide, and to prioritise ideas. Ultimately, that is a threat to the integrity of our shared reality, as it misleads some people with whom we share our community, making it impossible to understand each other or to agree anymore.

We seem to be evolving toward a civilisation increasingly governed not by physical constraints, but by informational ones. Energy, water, and land will continue to matter deeply, but or futures will be affected by failures to access and act upon high-quality information in time. The imperative will be to start asking the question: ‘What information would be most valuable to generate next?’ This would reorient science away from mere accumulation of information towards a more strategic production.

Moreover, based on this understanding, what we call ‘knowing’ is often just ‘assigning high probability and acting as if it were certain’. The most valuable information will revise the probabilities away from deeply held beliefs, which is the core of learning and the essence of intellectual humility. In this light, the scientific method emerges not merely as a tool for generating new information and testing hypotheses, but also as a kind of cognitive compass – a disciplined way of asking, ‘What should we all believe now, given this new piece of information?’ But even this compass may be weakened in today’s context, because if valuable information can shift beliefs and guide people to action, then it can also be suppressed, distorted, or even weaponised. A major ethical dilemma of our time will be how to protect the credible knowledge in a world where attention is heavily monetised and where manipulation with information can be far more profitable than spreading the truth.

In response, we may envisage that the roles of the scientist, the health professional, the policymaker, the journalist, the investor, and the informed citizen will evolve from mere users of information to curators, filters, validators, and amplifiers of information, too. Valuable information will continue to update our mental maps, so that we could generate and follow better ideas, make better decisions, save more lives, and understand our world with greater clarity. In this, success will favour those who find valuable information where the uncertainty was the greatest.

The CHNRI method illustrates the same principle: by crowdsourcing hundreds of expert judgments, it converts individual opinion, which is normally incommunicable and unscalable, into a dataset whose aggregate scores expose hidden consensus. Decision-makers gain a map showing which bets carry the highest expected return for the least risk, so their fear of being wrong can be discarded and financial flows unlocked. In the media example, the logic reverses: disinformation is negative-value information, because it expands uncertainty. It widens the probability distributions so broadly that rational action stalls, allowing vested interests to profit.

All five examples together suggest that the ‘architecture’ of valuable information can be understood through some of its inherent traits: relevance, *i.e.* it should influence an observer’s perception of the related beliefs or hypotheses; credibility, *i.e.* it needs to be trusted by an observer; and leverage, *i.e.* it should have the ability to influence an observer’s ideas and decisions decisively. Where all three criteria align, information assumes a ‘high value coefficient’, signifying the high likelihood of the expected reduction in costly error. Designing activities to increase this coefficient is the common interest of scientists, health professionals, policymakers, journalists, and investors.

When we extend this ‘architecture’ into the age of AI and information superabundance, the dominant constraint will no longer be how to collect information, but how verify and route it. Likewise, when valuable information is generated and used only by the minority in the society, social distribution of power will increase inequities. Therefore, access to high-value information, or at least to the means of producing and validating it, may become an important challenge in the future for the society as a whole. It may still be comforting that the winners – both scientific and societal – will not necessarily be those who simply acquire and own the most bytes of information. Success will follow those who consistently generate the tiny subset of bytes that change crucial prior knowledge and beliefs, doing so within trusted frameworks, robust enough to withstand disinformational noise.

Training both our machines and our minds not merely to detect patterns, but to discern which missing information, if produced, would unlock the greatest further learning or clearest path to collective action. It would lead to fastest and greatest progress. Not all information is created equal. Some data merely nudge our confidence, confirming what we already suspected. Other information causes major shifts, dramatically raising or collapsing the probability of a given belief. That is the kind of information that drives science forward. That information is the most valuable.
